# A new species of tiger beetle from southeastern Arizona and Mexico (Coleoptera, Carabidae, Cicindelini)

**DOI:** 10.3897/zookeys.464.8424

**Published:** 2014-12-16

**Authors:** Daniel P. Duran, Stephen J. Roman

**Affiliations:** 1 Department of Biodiversity, Earth and Environmental Sciences, Drexel University, 3245 Chestnut St., Philadelphia, PA 19104, USA; 2 5335 Oxbow Place, Champlin, MN 55316, USA

**Keywords:** Coleoptera, Cicindelini, *Cicindelidia*, new species, Arizona, Chiracahua Mountains, Mexico

## Abstract

A new tiger beetle species, *Cicindelidia melissa* Duran & Roman, **sp. n.**, of the tribe Cicindelini, is described from high elevation montane forests of southeastern Arizona and Mexico. It appears to be most closely related to *Cicindelidia nebuligera* (Bates) but is distinguished on the basis of multiple morphological characters and geographic range. The new species is also superficially similar to the widespread *Cicindelidia sedecimpunctata* (Klug), but distinguished on the basis of multiple morphological characters and habitat. Habitus, male and female reproductive structures, and known distribution map are presented.

## Introduction

The New World tiger beetle genus *Cicindelidia* Rivalier (1954) includes approximately 60 described species (Wiesner 1992) and is distributed from Canada south to Chile, reaching its highest diversity in Mexico and the southern United States. Members of the genus are diurnally active insect predators and are typically found in open or sparsely vegetated muddy, rocky, or sandy habitats. The majority of species inhabit areas that range from sea level to mid elevations, with only a few species known to occur at elevations above 2000 m. Herein we describe *Cicindelidia melissa* sp. n., an inhabitant of high elevation Ponderosa pine forests and discuss its hypothesized systematic placement within the genus.

## Methods

Specimens of a previously undescribed *Cicindelidia* had been collected over the past several decades by David Brzoska (Naples, FL), Ron Huber (Bloomington, MN), Walter Johnson (Minneapolis, MN) and John Stamatov (Armonk, NY) from a site in the Chiracahua Mountains of southeastern Arizona and from 29 localities in the Mexican states of Sonora, Chihuahua and Durango. Additional Chiracahua specimens were collected in 2009 by Eric Sangregorio and donated to the first author. In total the authors examined 153 specimens of the new species. Type material is deposited in the following institutional and private collections (acronyms used in the text are in parentheses): National Museum of Natural History, Smithsonian Institution, Washington, DC, USA (NMNH), Arizona State University Frank Hasbrouck Entomology Collection, Tempe, AZ (ASUHIC), Collection of David W. Brzoska, Naples, FL (DWBC), Ronald L. Huber Collection, Bloomington, MN (RLHC), Collection of Walter N. Johnson, MN (WNJC), Collection of Daniel P. Duran, Philadelphia, PA (DPDC), Collection of John Stam atov, Armonk, NY (JSC). Specimens were compared to material of all putative close relatives, including *Cicindelidia sedecimpunctata* and it’s subordinate taxa *mellyi* and *sallei*, *Cicindelidia flohri*, and *Cicindelidia nebuligera*.

Images of the dorsal, lateral, and frontal habitus and elytral apex were captured using a Canon EOS 7D attached to a Visionary Digital Imaging System (Visionary Digital, Palmyra, VA). Images were then montaged and edited using Adobe Photoshop. Genitalia were extracted, manually cleaned with minuten pins and 10% KOH solution, and placed on glycerin slide mounts for observation and imaging. Scale bars were calibrated with an ocular micrometer using SPOT Advanced software on the images of the genitalia, which were taken with a digital camera attached to a Nikon SMZ1500 dissecting microscope. The final digital images were processed using Adobe Photoshop CS6. The distribution map was created with Quantum GIS Version 1.4.0.

Body measurements are defined as in Duran and Moravek (2013) and are as follows. The total body length excludes the labrum and is measured as the distance from the anterior margin of the clypeus to the elytral apex, including the sutural spine. The width of the pronotum is measured to include the lateral margins of the proepisterna. The width of the head is measured as the distance between the outer margins of the eyes.

## Systematics

### Cicindelidia melissa

Taxon classificationAnimaliaColeopteraCarabidae

Duran & Roman sp. n.

http://zoobank.org/E474385B-6B10-474A-A705-1F340C0B8FD5

Figs 2 , 3 , 4 , 5 , 6 , 7 , 8 , 9 , 10

#### Type material.

HOLOTYPE: ♂, “USA, Arizona / Cochise Co / Barfoot Park (31.910, -109.273) D. Brzoska Aug 4, 2012” (USNM). ALLOTYPE: ♀, “USA, Arizona / Cochise Co / Barfoot Park (31.910, -109.273) D. Brzoska, Aug 4, 2012”(USNM).

PARATYPES: 1 ♂, USA, Portal Ariz / Barfoot Park / 08-VIII-1957 8000ft. / leg J.R.Beer. 13 ♂♂, 9 ♀♀, same label data as Holotype. 1 ♂, 3 ♀♀, USA, Arizona / Cochise Co / Barfoot Park (31.910, -109.273) E. Sangregorio, Aug 4, 2009. 1 ♂, 1 ♀, MEXICO, Chihuahua /Hwy.25 km157,. 5. S. Cusarare (27.332, -107.005) D. Brzoska, July 29, 2005. 1 ♂, 1 ♀, MEXICO, Chihuahua / Road to Z.A. Conjunio Anasezi / 1.6mi. S.,. 09mi. W-Madera (29.172, -108.173) D. Brzoska, July 11,1997. 1 ♂, 1 ♀, MEXICO, Chihuahua / Creel, Divisidero Rd, 0.5m S Divisidero (29.528, -107.830) D. Brzoska, July 21, 2005. 1 ♂, 1 ♀, MEXICO, Chihuahua / Hwy. 26, (Road to Topia), km 38 (25.063, -105.655) D. Brzoska, July 24, 1997. 2 ♂♂, 2 ♀♀, MEXICO, Chihuahua / Chi Hwy. 25, E. of/ Guachochi-km104 / 26-VII-2005 R.L. Huber. 1 ♂, MEXICO; Chihuahua / Chi Hy 25 KM 157/ 20-VII-2005 R.L. Huber. 2 ♂♂, MEXICO, Chihuahua / KM 25 Road to Batopilas / Quirare Village / 26-VII-2005 R.L. Huber. 3 ♂♂, MEXICO, Chihuahua / km 18, N of Batopilas / Road to Creel / 21-VII-2005 R.L. Huber. 4 ♂♂, 9 ♀♀, MEXICO, Chihuahua / Chi Hwy 25 KM 157 / S of Cusarare / 26-VII-2005 R.L. Huber. 3 ♂♂, 4 ♀♀, MEXICO, Chihuahua / Ej.Guadalupe Victoria / 9.8mi W. San Jose Babicora / on Hwy 180, near km 14 /10-VII-1992 R.L. Huber. 1 ♀, MEXICO, Chihuahua / Hwy 180 9.1 mi W / San Jose Bibicora / 13-VIII-1989 R.L. Huber. 1 ♀, MX / Chihuahua; Madera / June 1966 leg B. Rotger. 1 ♂, MX Chihuahua / 20mi S la Junta / 29-Vi-1989 D.B. Thomas and J.C. Burne.

5 ♂♂, 2 ♀♀, MEX, Chihuahua /Road to Divisadero / (27'38.72N, 107'46.27W) / July 2, 1997 R.L. Huber. 5 ♂♂, 6 ♀♀, MEX, Chihuahua / .5mi S Divisadero / (27'31.69N, 107'49.80W) / July12.1997, R.L. Huber. 2 ♂♂, 2 ♀♀, MEX, Chihuahua / S. of Creel / (27'41.64N, 107'35.14W) / July 12, 1997 R.L. Huber. 1 ♂, 2 ♀♀, MEX, Chihuahua / Ejido Guadalupe / (29'12.86N, 107'52.81W) / July 11, 1997 R.L. Huber. 1 ♂, 2 ♀♀, MEX, Chihuahua / 2mi SW Madera / Rd to Sirupa / (29'10.21N, 108'09.64W) / July11.1997 R.L. Huber. 2 ♂♂, 1 ♀, MEX, Chihuahua / 2.5mi W Madera / Rd to Huapaca Archaeo site / (29'10.29N, 108'10.40W) / July 11, 1997, R.L. Huber. 1 ♂, 1 ♀ MEX, Chihuahua / N of Madera / HWY 11, KM13.5 / (29'18.8N, 108'08.4W) / July 25,2008 R.L. Huber.

1 ♀, MEX, Chihuahua / Ejido Guadalupe Victoria / HWY 10, km 13.5 / (29'12.9N, 107'52.3W), 2300m / Aug 06, 2008 R.L. Huber. 1 ♂, MEX, Chihuahua / Creel N. HWY 25 KM72 / (27'51.7N, 107'34.7W), 2362m / Aug 05, 2008 R.L. Huber. 1 ♂, MEX, Chihuahua / San Juanito, NNW / on Chi HWY110 KM5.5 / (27'58.9N, 107'31.9W), 2440m / Aug 05.2008 R.L. Huber. 1 ♀, MEX, Chihuahua / 5KM S Madera, Rd to Sirupa / (29'09.0N, 108'10.6W), 2230m / July 25, 2008 R L Huber. 1 ♂, MX Chihuahua / Hwy 180 9.1 mi W / San Jose Bibicora / 13-VIII -1989 R.L. Huber. 2 ♂♂, 3 ♀♀, Durango Mexico / Lagoya de Golondrines / July 23, 1997 / Walter N Johnson. 2 ♂♂, 1 ♀, Durango Mexico / Rancho Chapultepec / July 23, 1997 / Walter N Johnson. 3 ♂♂, 1♀ Durango Mexico / Los Altares, HWY 26 / July 24, 1997 / Walter N Johnson. 3 ♂♂, 1 ♀, Durango Mexico / Los Ranes / July 23, 1997 / Walter N Johnson. 1 ♀, Durango Mexico / Los Ranes / July 24, 1997 / Walter N Johnson. 2 ♂♂, Durango Mexico / 2.5mi E. Los Ranes / July 23, 1997 / Walter N Johnson. 9 ♂♂, 5 ♀♀, MEXICO Chihuahua / Ejodo Guadalupe Victoria / 9.8 mi W S.J. Bibicora / 10-VII-1992 J.Stamatov. 4 ♂♂, 6 ♀♀, MEXICO Chihuahua / Hy 25, km157.5 2172m / S of Cusarare (27°19.9 107°30.3) / July-26-2005 Coll: J. Stamatov. 1 ♂, 3 ♀♀, MEXICO Durango / 13.5 mi E of Canelas / 15-VII-1997 / Coll: J. Stamatov.

All type specimens labelled: HOLOTYPE, ALLOTYPE or PARATYPE, respectively.

#### Diagnosis.

**Figure 1.**
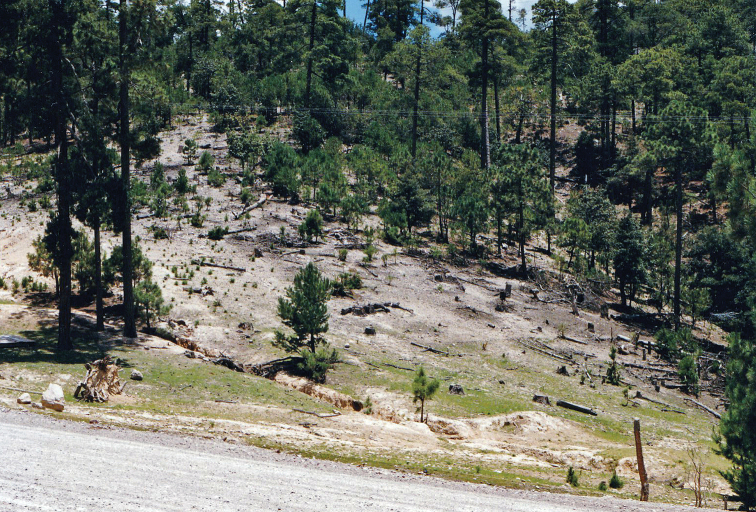
Habitat of *Cicindelidia melissa*, Durango, Mexico. Photo by Walter Johnson.

This species can be distinguished from all other similar *Cicindelidia* by its dark green-violet abdominal venter with the two apical segments dull orange or orange-brown, a brassy-cupreous head and pronotum with metallic blue reflections in sulci, small shallow subsutural foveae present in most individuals, and microserrate elytral apices. It inhabits rocky upland soils in ponderosa pine forests above 2000 m (Fig. 1). *Cicindelidia sedecimpunctata* (Klug, 1834) has an entirely orange-red to orange-brown abdominal venter, a more uniform dull brown dorsal coloration, and lacks apparent subsutural foveae. It also differs from the new species by inhabiting muddy ground at nearly any elevation. *Cicindelidia nebuligera* (Bates, 1890) has dark elytral infuscations that surround the middle band, and lacks elytral apical microserrations. It may be found in similar habitats, but is apparently allopatric with the new species and does not appear to be restricted to elevations above 2000 m.

#### Description.

**Figure 2.**
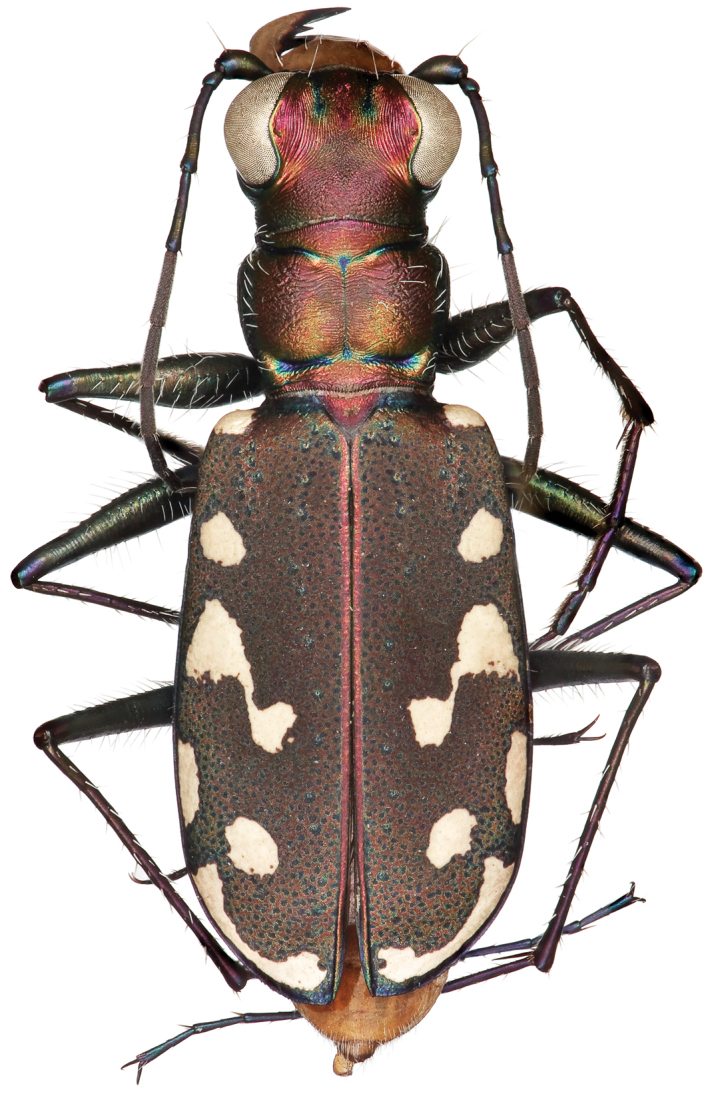
Dorsal habitus of male (holotype).

**Figure 3.**
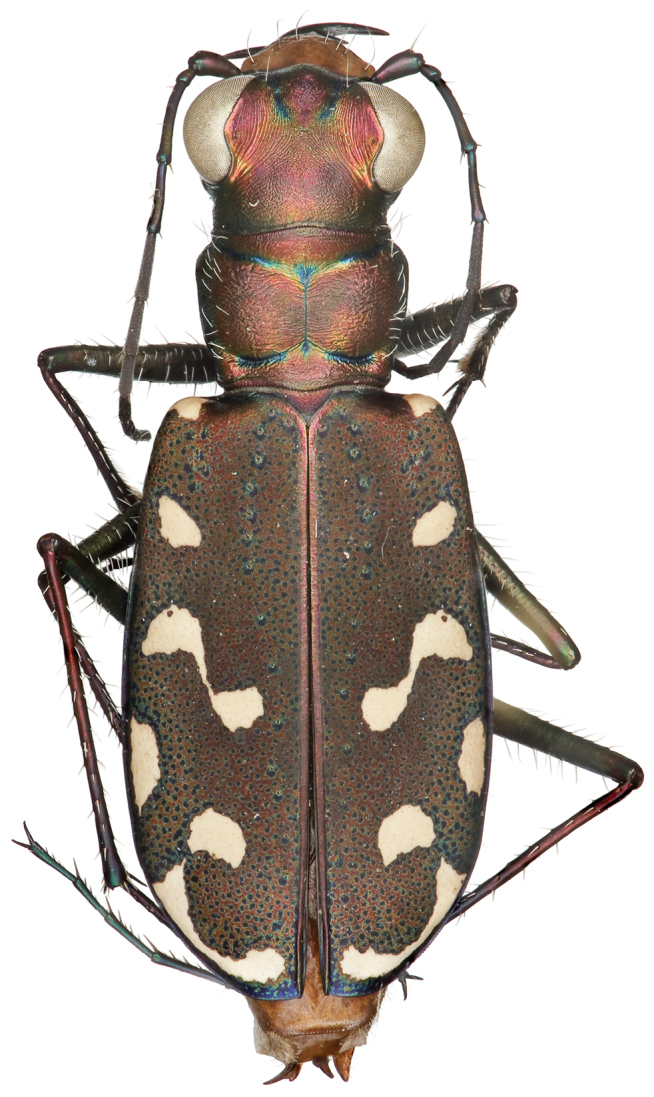
Dorsal habitus of female (allotype).

**Figure 4.**
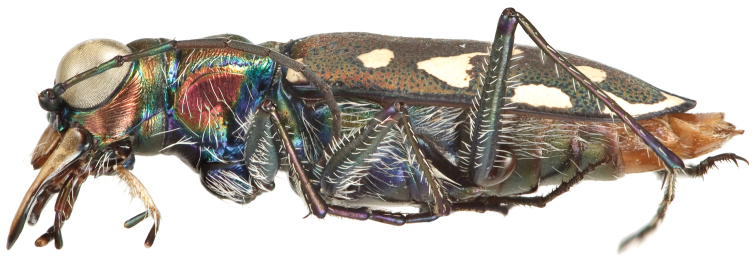
Lateral habitus of male (holotype).

**Figure 5.**
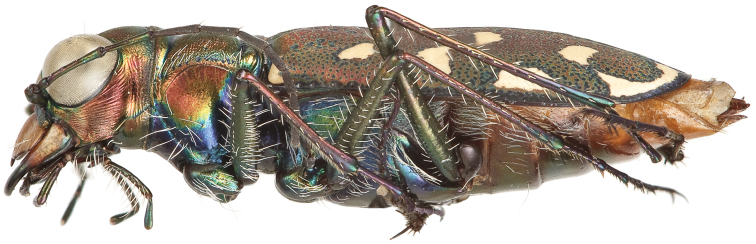
Lateral habitus of female (allotype).

**Figure 6.**
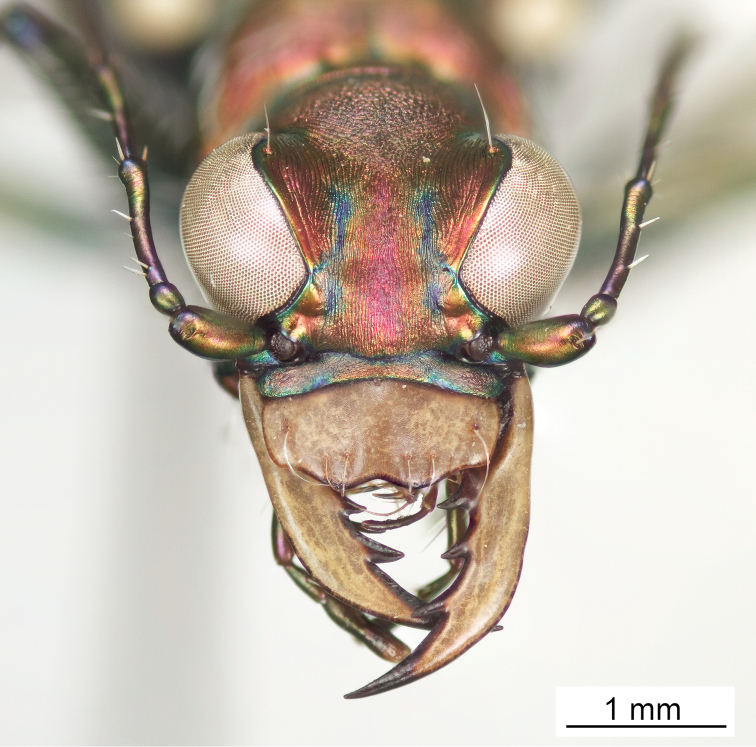
Frontal habitus of male (holotype).

**Figure 7.**
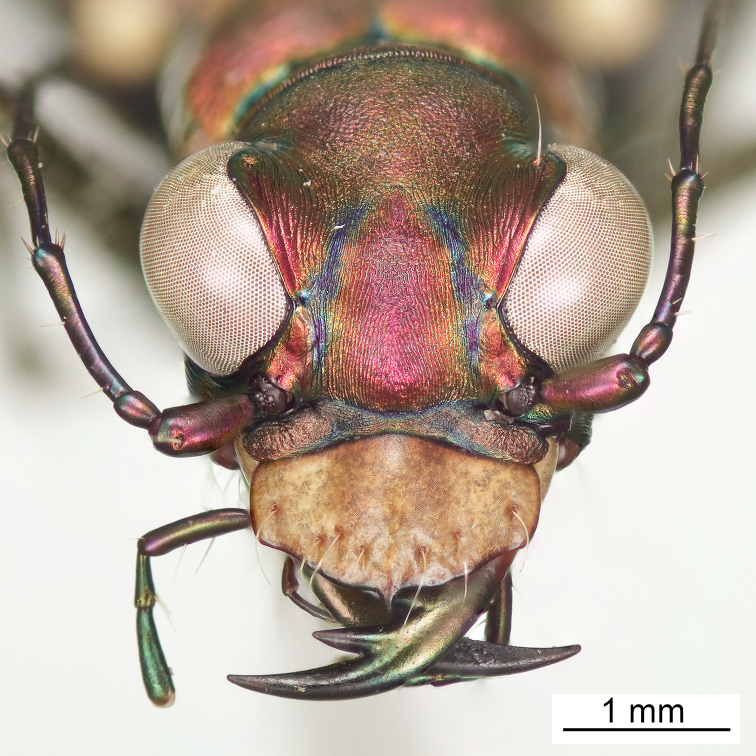
Frontal habitus of female (allotype).

Small to medium sized *Cicindelidia*. Body (Figs 2–5) length 7.90–10.50 mm, mean ♀ 9.7 mm, mean ♂ 9.0 mm. Head (Figs 6–7) slightly wider than pronotum, width 2.3–2.7 mm, brassy-cupreous red with metallic blue and cupreous reflections present in sulci, all head portions glabrous except for 2 supraorbital setae next to each eye. Frons concave in median area especially in male, bulging towards slightly convex near anterior margin, clearly delimited from clypeus, gradually blending into vertex. Frons surface with distinct longitudinal striae especially in lateral areas bordering eyes, vermiculate-striate in median area. Vertex dark brassy colored, slightly convex, with surface indistinctly finely vermiculate, posterior areas with cupreous-olive lustre. Genae bright polished copper with deep longitudinal striae abruptly ending at border of vertex. Clypeus cupreous blending to blue along borders, irregularly wrinkled to finely vermiculate. Labrum with 6 setae, ochre-testaceous with a thin dark brown to black border; female labrum rather long, length 0.60–0.90 mm, width 1.3–1.6 mm, with single median tooth; male labrum short to medium, length 0.45–0.80 mm, width 1.2–1.7 mm, shape varies from nearly straight across anterior edge with only slightly protruding median tooth to an unusual slightly notched median edge (see holotype). Mandibles medium-sized, ochraceous in male, dark ochraceous with metallic gold, green and black reflections in female, teeth of both sexes dark testaceous along edges. Maxillary palpi dark testaceous with metallic reflections; apical segment usually darker than sub-apical segment. Labial palpi in male ivory to pale yellow-ochre in male with dark metallic green to violet apical segment, in female entirely dark testaceous with metallic reflections throughout. Antennae normal length, reaching humerus to basal third of elytron, slightly longer in male than female; scape dark testaceous to black with metallic reflections of cupreous, gold, and violet, with a single apical seta; pedicel dark testaceous with metallic reflections of cupreous, gold, and violet, lacking any setae; flagellum dark testaceous, antennomeres 3–4 with metallic cupreous and violet reflections, with ring of apical setae and additional sparse setae throughout, antennomeres 5–11 dull textured without metallic reflections and possessing erect setae in apical rings only, covered with fine pubescence throughout.

**Figure 8.**
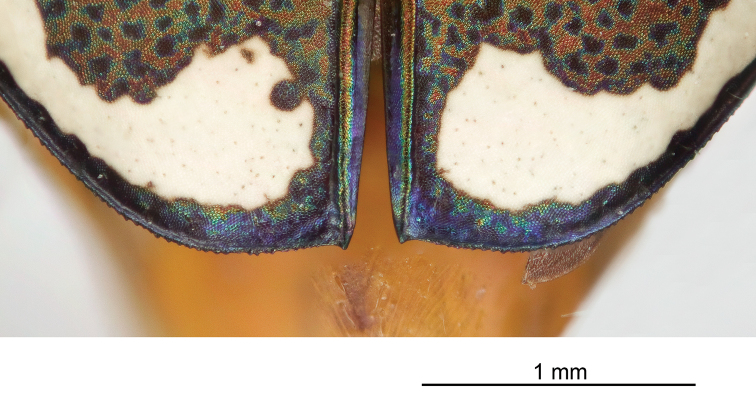
Elytral apex, showing microserrations and apical spine.

Thorax. Pronotum 1.70–2.50 mm in width, slightly polished with metallic finish, brassy-cupreous with metallic blue or blue-green sulci, slightly wider than long, nearly trapezoidal in shape and widest near anterior margin, width to length ratio 1.2 to 1.3, setae sparse and present along lateral third of dorsal surface; disc finely rugose to vermiculate with thin but distinct median line and deeply impressed sulci; notopleural sutures clearly defined, not visible from dorsal view; proepisternum bright polished copper with gold and green reflections more ventrally, abruptly transitioning to blue-violet on ventral third and posterior third, in male setae present throughout surface of proepisternum, but in female setae are typically sparsely present only along ventral third and along anterior margin; all other ventral segments of thorax are glabrous, dark blue-violet to black with greenish reflections. Elytra elongate, 5.1–6.7 mm length, shape similar in both sexes, but slightly wider in female, especially toward apical third; sutural spine small to nearly absent, fine microserrations present on elytral apices (Fig. 8), extremely fine to nearly indistinct in some individuals; elytral dorsal surface relatively flat, not markedly convex, texture dull throughout with slight metallic sheen near pronotal base in a some individuals, elytral coloration mostly a dull cupreous brown color, under magnification this color is comprised of the pointillistic mixing of mostly cupreous ground color with many small patches of dark blue-violet bordered in green; subsutural foveae are present, but nearly indistinct in a small percentage of individuals; elytral maculations white, and consist of a small humeral and posthumeral spot, a moderately short middle band which does not touch the margin and with “knee” and “foot” regions connected with a thin but complete line, an isolated marginal spot between the middle band and apical lunule, and an apical lunule comprised of a subapical spot that is broken from the thin apical line; epipleura dark blue-violet to black.

Legs. Procoxae and mesocoxae dark metallic green to black, covered in dense setae; metacoxae dark metallic green to blue-violet to black, with a single apical setae present; trochanters glabrous, dark green to violet-black; femora dark metallic green with blue-violet reflections near the insertion of the tibia, femoral surface with rows of erect white setae dorsally and ventrally; tibiae violet to dark cupreous with dark green reflections near the apices, clothed with white setae that are sparser and shorter than those of the femora; tarsi violet with blue reflections dorsally, first three dilated protarsomeres in male with dense greyish-white setal pad.

Abdomen. Ventrites 1–4 dark violet with strong metallic greenish reflections throughout most surfaces, dark orange to testaceous coloration along lateral edges in some individuals, setae present mostly along lateral third of each ventrite; ventrites 5–6 orange to dark orange-testaceous throughout, setae present along lateral margins, but often abraded.

**Figure 9.**
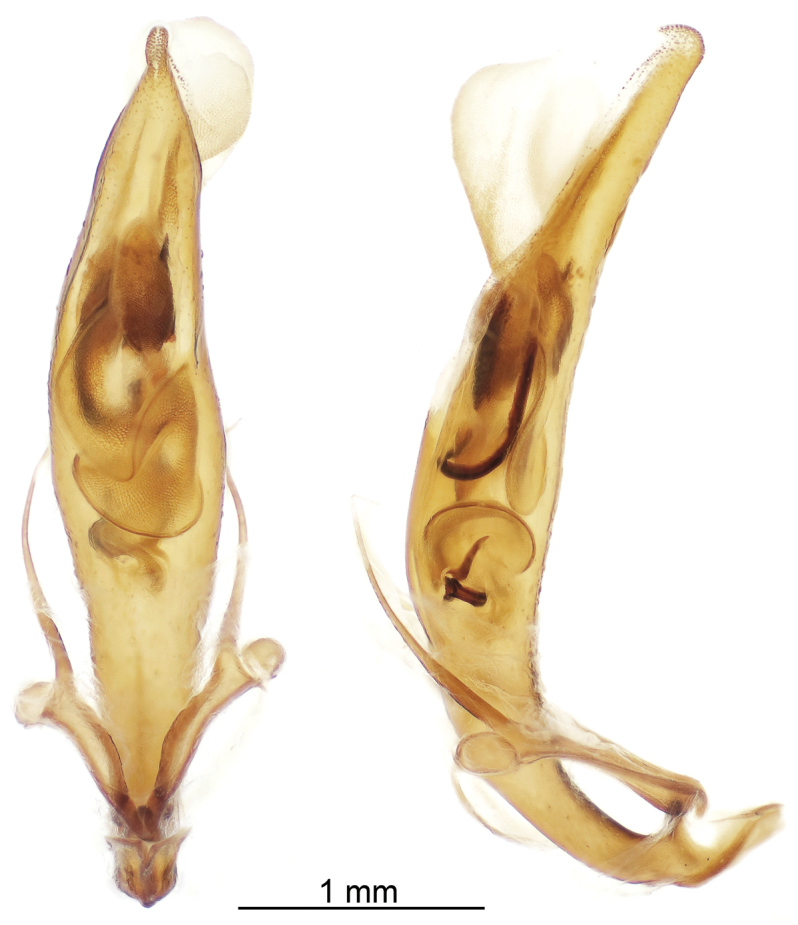
Cleared aedeagus in ventral and lateral (left side) views.

**Figure 10.**
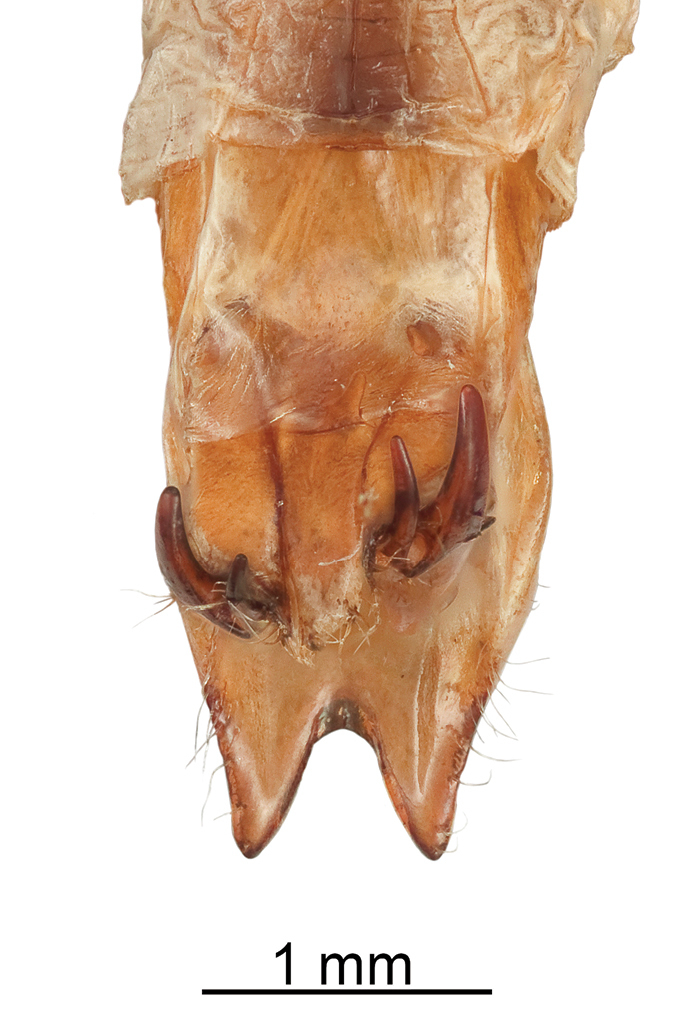
Ovipositor in ventral view.

Reproductive structures. Aedeagus (Fig. 9) elongate, widest in middle, length 3.40–3.60 mm, width 0.65–0.75 mm, slightly arcuate in ventral view, apical portion produced into a narrow tip with a slight hooklike projection; internal sac and sclerites prominent in cleared aedeagus and visible both ventrally and laterally. Ovipositor (Fig. 10) deeply notched and possessing two heavily sclerotized bifurcated hooks ventrally, setae present especially along lateral margins and near base of hooks.

#### Etymology.

This new *Cicindelidia* is named after the first author’s wife, Melissa, for her constant support, love, and friendship.

#### Distribution and habitat.

*Cicindelidia melissa* is currently known from northwestern Durango, western Chihuahua, eastern Sonora, and southeastern Arizona. All known occurrences are from forested hillsides and trails above 2000 m. Typical habitats contain rocky substrates derived from limestone and/or rhyolite, with forest cover generally dominated by Ponderosa pine. This species is mostly active following monsoon rains, but frequents upland areas and is not closely associated with muddy or riparian microhabitats.

**Figure 11.**
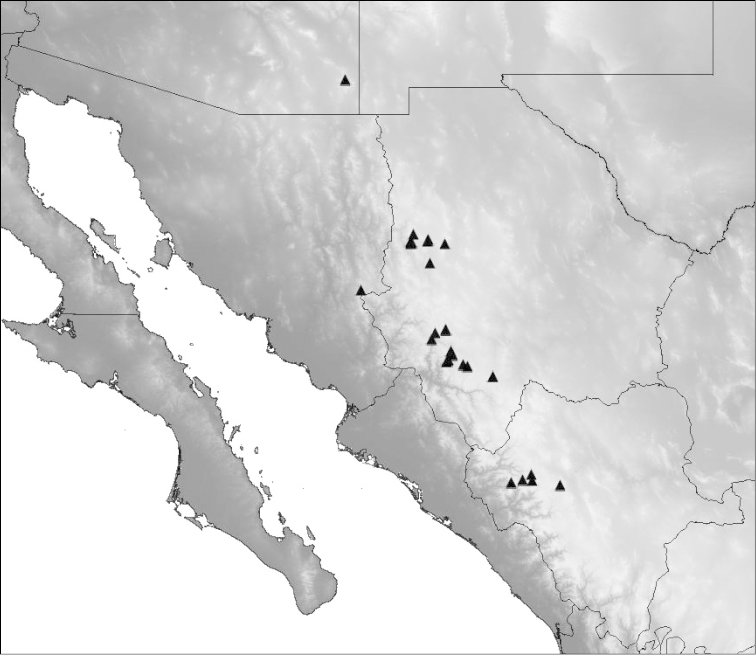
Distribution map of the known localities for *Cicindelidia melissa*. Lines indicate political boundaries of states in Mexico and the United States. Shading indicates topographical relief.

## Discussion

Given the superficial similarity to *Cicindelidia sedecimpunctata* in dorsal habitus, *Cicindelidia melissa* may have been overlooked and assumed to be a form of that widely distributed species. However, despite the general resemblance, multiple diagnostic morphological characters exist, as discussed above. Previous authors acknowledged that additional cryptic species may be present in the *Cicindelidia sedecimpunctata/rufiventris* group (Cazier 1954, Murray 1980), but the taxonomy of the group has not been revisited since. The relatively small number of available specimens of *Cicindelidia melissa* in museums is likely due to its occurrence in less-accessible geographic areas and in a habitat that is less visited by most tiger beetle collectors.

It is interesting to note that the ecological differences between *Cicindelidia melissa* and *Cicindelidia sedecimpunctata* are stark, and habitat alone separates the two species in almost all cases. Tiger beetle taxonomy has relied nearly exclusively on fixed morphological characters to date, yet we believe that this present example underscores the importance of habitat and ecological factors that may not be apparent when comparing dead specimens of tiger beetles. Given the ecological and morphological similarities and apparently allopatric ranges, we propose that *Cicindelidia melissa* and *Cicindelidia nebuligera* are most closely related. Increasingly, higher-level and species-level phylogenies are based on molecular data, in part or entirely, and recent authors have examined relationships of Nearctic tiger beetles (Vogler et al 2005), although Mexican species were not as well represented. The authors of this description are conducting a thorough revision of *Cicindelidia* using a combination of molecular, morphological, and ecological characters, and this species description is the first of a series of papers on the group.

## Supplementary Material

XML Treatment for Cicindelidia melissa
